# Effectiveness and safety of acupuncture combined with hot compress in the treatment of obese adolescents with insulin resistance: A protocol for systematic review and meta-analysis

**DOI:** 10.1097/MD.0000000000032235

**Published:** 2022-12-23

**Authors:** Xiaochao Gang, Yuxing Tai, Zhenxiang Xiao, Xiaobo Jiang, Dilnur Barat, Tianjiao Gao, Yiran Han, Jie Liu, Chongwen Zhong, Shaotao Chen, Mingjun Liu

**Affiliations:** a Changchun University of Chinese Medicine, Changchun, China; b Third Affiliated Clinical Hospital to Changchun University of Chinese Medicine, Changchun, China; c Acupuncture and Massage Center of the Third Affiliated Clinical Hospital of Changchun University of Chinese Medicine, Changchun, China.

**Keywords:** acupuncture, adolescents, hot compress, obesity, protocol

## Abstract

In recent years, the incidence of obesity patients has become younger and younger, and adolescents are gradually becoming one of the groups with a high incidence of obesity. Although several systematic reviews and clinical trials suggest that acupuncture and warm compresses may be effective in the treatment of obesity, the effectiveness and safety of acupuncture combined with warm compresses in the treatment of obesity insulin resistance (IR) in adolescents have not been systematically reviewed.

**Methods:**

The search language of this study is Chinese and English, and the data of Medline, PubMed, Embase, Cochrane Web of Science, China Biomedical Literature Database, Central Controlled Trial Registration Center, and China Scientific Journal Database were searched for this study respectively, from the date of creation of the above data to December 2022. Randomized controlled trials of acupuncture combined with warm compresses in adolescents with obese IR were included in this review. Main outcome measures were body mass index, waist circumference, hip circumference, waist-hip ratio, fasting blood glucose, glycosylated hemoglobin, IR index, body fat content, blood lipid level and blood pressure, etc. In addition, we manually retrieved other resources, including reference lists of identified publications, conference articles and gray literature.

**Results:**

This study will provide more clinical treatment ideas and options for adolescent obese IR patients.

**Conclusion:**

The purpose of this study is to summarize and evaluate the efficacy and safety of acupuncture combined with hot compress in treating obesity IR in adolescents from clinical trials.

## 1. Introduction

Obesity is a serious threat to human health and can cause insulin resistance (IR). In recent years, the number of obese adolescents with IR is increasing year by year. Although the obesity rate of adolescents is lower than that of adults, its growth rate is higher than that of adults.^[1,2]^ As IR is an important pathogenesis of many diseases related to endocrine and metabolic disorders, obesity IR not only affects the normal development and daily life of teenagers, but also causes a series of diseases related to metabolic disorders, such as cardiovascular diseases, digestive diseases, skeletal diseases and so on.^[3–5]^ IR is a serious threat to the health of teenagers not only from the physiological aspect, but also from the psychological aspect. If we don’t intervene, it will lead to more adult obese patients with IR in the future.

At present, the commonly used treatments for obese patients with IR include: lifestyle education and guidance, drug treatment, surgical treatment, etc.^[6]^ Roux-en-Y gastric bypass and sleeve gastrectomy were used during the operation.^[7,8]^ However, teenagers’ compliance is generally poor, and the educational guidance effect is not ideal. Oral administration of drugs may cause side effects. There is a risk of complications in the operation. The common complications are wound infection, pulmonary embolism, abdominal hernia, small intestinal obstruction, etc. Therefore, there is an urgent need to find more safe and effective methods to treat obesity IR in adolescents. Acupuncture and hot compress can play a certain role in treating this disease.

Acupuncture and hot compress are both external treatments of traditional Chinese medicine, and the combination of acupuncture and hot compress has proved to be effective in treating obesity IR. However, at present, there is no systematic review and meta-analysis on the efficacy and safety of acupuncture combined with hot compress in the treatment of obesity IR in adolescents. Therefore, through the supplement of this study, we hope to provide more safe and effective treatment schemes for clinical treatment of obesity IR.

## 2. Methods and analysis

This systematic review has been registered in the PROSPERO network (No. CRD42022367970). All steps of this systematic review will be performed according to the Cochrane Handbook (5.2.0).^[9]^

### 2.1. Inclusion criteria

#### 2.1..1. Types of studies.

All the literatures included in this research and analysis belong to random clinical research test type. In order to ensure the accuracy of the results, meta-analysis, animal experiment, literature review and case literature were excluded.

#### 2.1..2. Types of participants.

The included patients should be adolescents aged <18 years and >12 years, with body mass index (BMI) ≥24 kg/m^2^, regardless of gender, nationality, region or nationality. Before inclusion, patients need to be excluded from major diseases such as cancer, taking drugs, pregnancy and other factors, and all included people need to be diagnosed as obese IR.

#### 2.1..3. Types of interventions and comparisons.

In the treatment group, adolescent obese patients with IR received intervention methods including acupuncture combined with hot compress, and the time and frequency were not limited. Patients in the control group can take Western medicine, Chinese patent medicine or placebo orally, massage, cupping, moxibustion and so on.

#### 2.1..4. Types of outcome measures.

The main measurements will be body mass index, waist circumference, hip circumference, waist-hip ratio, fasting blood glucose, glycosylated hemoglobin, and IR index. The body fat content, blood lipid level and blood pressure, etc will also be included in the evaluation scope.

### 2.2. Search strategy

The searched databases include English and Chinese, including PubMed, Embase database, Wanfang database, Cochrane Central Registry of Controlled Trials, Knowledge Infrastructure of National Library of China, China Clinical Trials Registry database, China Scientific Journal database, and other related research materials. In the process of searching, we will also screen the list of references of related papers, and have obtained more data sources to support this research. The time range of retrieval is from the date of creation of the above database to December 2023. Keywords include “acute,” “Hot Compress,” “Obese,” “Adolescents,” “Insulin Resistance” and so on. PubMed retrieval strategy will be briefly provided as an example in Table 1.

**Table 1:** Search strategy used in Pubmed database.

Number	Search terms
#1	Obesity (All files)
#2	Adolescent (All files)
#3	Insulin resistance (All files)
#4	Obese adolescents with insulin resistance (All files)
#5	#1 AND #2 AND #3 OR #4
#6	Acupuncture (All files)
#7	Acupuncture treatment (All files)
#8	Traditional Chinese medicine acupuncture (All files)
#9	Hot Compress (All files)
#10	Warm compress (All files)
#11	Apply a heat pack (All files)
#12	Apply a heat pack (All files)
#13	Acupuncture combined with Hot Compress (All files)
#14	#6 OR #7 OR #8 AND #9 OR #10 OR #11 OR #12 AND #13
#15	Randomized controlled trial (All files)
#16	Controlled clinical trial (All files)
#17	Randomly (All files)
#18	Randomized (All files)
#19	Placebo (All files)
#20	Double-blind method (All files)
#21	Single blind method (All files)
#22	Trials (All files)
#23	#15 OR #16-22
#24	#5 AND #14AND #23

### 2.3. Study screening

**Figure 1.**
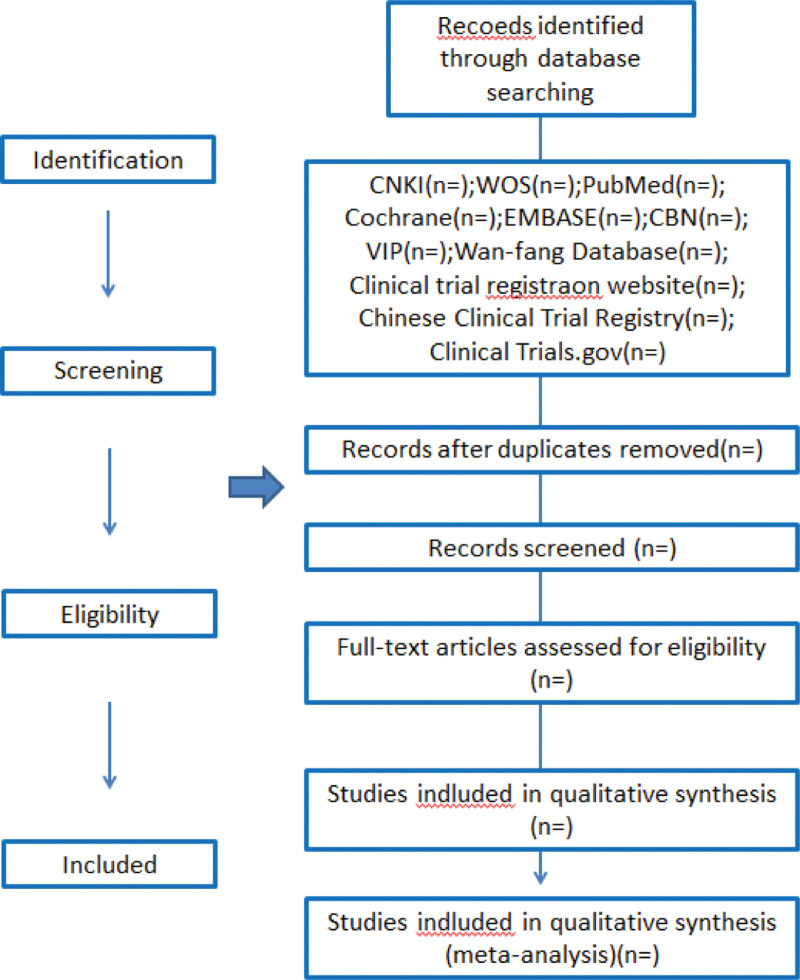
Flow diagram of study selection process.

After the preliminary data is extracted, it is screened to eliminate duplicate content. Two team members independently screen titles and abstracts according to the qualification criteria, and build an extraction list that meets the criteria. When there is disagreement in the data collection process, the team will make a decision in the form of group discussion. The process of identifying and selecting documents is shown in Figure 1.

### 2.4. Data extraction

Two researchers in the research team further extracted qualified data, including trial design, patient’s gender, age, course of disease, intervention measures, intervention time, intervention duration, intervention frequency, intervention results, patient feedback, adverse reactions after intervention, etc. If there is a difference, another researcher (MJL) will make a decision on the part.

### 2.5. Statistical analysis

The flow chart is created by PRISMA scale and Review Manager version 5.3. The meta-analysis software was Revman 5.3 and Stata 16. The weighted average difference uses 95% confidence intervals to calculate continuous variables. The measurement and evaluation of heterogeneity in this study are completed by using Q test and *I*^2^ measurement. During the research process, researchers explored the root causes of heterogeneity through sensitivity analysis. In case of serious heterogeneity, check whether the data are correct again, such as whether there are errors in the analysis unit, or properly exclude a small number of related studies on possible sources of heterogeneity, so as to effectively reduce heterogeneity. When the heterogeneity is not significant (*P* ≥ .10 or *I*^2^ < 50%), the combined effect is analyzed by the fixed effect model, while when the heterogeneity is statistically significant (*I*^2^ ≥ 50% or *P* < .10), the random effect model studies the cause analysis of heterogeneity through subgroups. The research results are illustrated by forest map.

### 2.6. Assessment of heterogeneity

If >10 articles are included in the meta-analysis, the researcher will draw and analyze the funnel chart by using Revman 5.3, and observe whether the funnel chart is symmetrical, so as to judge whether the evaluation results are biased. There are 6 main types of bias: random sequence generation, distribution concealment, participant and individual blindness, outcome evaluation blindness, incomplete outcome data, selective reports and other bias sources. The quality of the evidence involved in the study is divided into 4 grades: high, medium, low and polar. Disagreements will be decided after discussion by the research team.

### 2.7. Sensitivity analysis and grading of evidence quality

In order to clarify the influence of individual research bias in the overall research results, the research team will judge and make decisions on the sensitive content in the research review process by analyzing the research method quality, sample size and results.

### 2.8. Subgroup analysis

We will consider subgroups such as jurisdiction, clinic type, and location (rural/urban).

## 3. Discussion

Obesity IR is one of the main mechanisms leading to obesity, and chronic low-grade inflammation in obese patients and also promotes IR, so obesity and IR often coexist. Obese IR is also associated with adipocyte dysfunction and macrophage infiltration. In the obese state, fat cells become larger in size, and immune cells such as macrophages are activated after entering adipose tissue, secreting a large number of inflammatory cytokines, thereby causing chronic low-grade inflammation around obese patients.^[10]^ As a complementary and alternative therapy, acupuncture combined with hot compress in the treatment has been proved to have good improvement and therapeutic effect through a large number of clinical trials, so in this study, we focus on the efficacy and safety of acupuncture combined with hot compress for the clinical treatment of adolescent obesity, in order to provide clinicians and patients with more choices to treat obesity.

## Author contributions

**Conceptualization:** Xiaochao Gang, Yiran Han.

**Data curation:** Yiran Han, Tianjiao Gao.

**Formal analysis:** Shaotao Chen, Yuxing Tai.

**Methodology:** Chongwen Zhong, Shaotao Chen.

**Software:** Zhenxiang Xiao, Dilnur Barat, Xiaobo Jiang, Jie Liu.

**Supervision:** Mingjun Liu.

**Writing – original draft:** Xiaochao Gang, Yuxing Tai.

**Writing – review & editing:** Xiaochao Gang, Mingjun Liu.
